# Resource Colimitation Drives Competition Between Phytoplankton and Bacteria in the Southern Ocean

**DOI:** 10.1029/2020GL088369

**Published:** 2021-01-12

**Authors:** Lavenia Ratnarajah, Stéphane Blain, Philip W. Boyd, Marion Fourquez, Ingrid Obernosterer, Alessandro Tagliabue

**Affiliations:** ^1^ Department of Earth Ocean and Ecological Sciences School of Environmental Sciences University of Liverpool Liverpool UK; ^2^ Sorbonne Université CNRS Laboratoire d'Océanographie Microbienne (LOMIC) Observatoire Océanologique de Banyuls Banyuls sur mer France; ^3^ Institute for Marine and Antarctic Studies University of Tasmania Hobart TAS Australia; ^4^ Aix Marseille Univ. Universite de Toulon CNRS IRD MIO UM 110 Marseille France

**Keywords:** bacteria, competition, iron, phytoplankton, Southern Ocean

## Abstract

Across the Southern Ocean, phytoplankton growth is governed by iron and light, while bacterial growth is regulated by iron and labile dissolved organic carbon (LDOC). We use a mechanistic model to examine how competition for iron between phytoplankton and bacteria responds to changes in iron, light, and LDOC. Consistent with experimental evidence, increasing iron and light encourages phytoplankton dominance, while increasing LDOC and decreasing light favors bacterial dominance. Under elevated LDOC, bacteria can outcompete phytoplankton for iron, most easily under lower iron. Simulations reveal that bacteria are major iron consumers and suggest that luxury storage plays a key role in competitive iron uptake. Under seasonal conditions typical of the Southern Ocean, sources of LDOC besides phytoplankton exudation modulate the strength of competitive interactions. Continued investigations on the competitive fitness of bacteria in driving changes in primary production in iron‐limited systems will be invaluable in refining these results.

## Key Points

- Competition for iron between phytoplankton and bacteria is modulated by the colimiting effects of light and dissolved organic carbon
- When enough labile dissolved organic carbon is present, bacteria can outcompete phytoplankton for iron
- Phytoplankton exudation may be insufficient to stimulate bacterial dominance in the absence of other dissolved organic carbon sources

## 1 Introduction

Phytoplankton growth in large areas of the Southern Ocean is regulated by the micronutrient iron (Fe) (Blain et al., 2007; Boyd et al., 2007; Martin et al., 1994), with seasonal colimitation by light (Mitchell et al., 1991; Strzepek et al., 2012) due to the deep mixed layers and ice cover that are prevalent in early spring, autumn, and winter. The low rates of primary production and large‐scale upwelling of deep water containing low dissolved organic carbon (DOC) concentrations lead to some of the lowest surface DOC concentrations in the global ocean (∼40–50 µM, Hansell et al., 2012). While low surface dissolved Fe and DOC is thought to restrict heterotrophic bacterial (hereafter bacteria) growth in the Southern Ocean (Church et al., 2000; Obernosterer et al., 2015), studies from Fe‐limited regions have demonstrated that bacteria may also be significant consumers of dissolved Fe and can exhibit greater cellular Fe quotas than phytoplankton (Boyd et al., 2012; Fourquez et al., 2015, 2020; Tortell et al., 1996, 1999). High bacterial Fe demand and uptake rates compared to phytoplankton suggest that bacteria could be significant competitors for dissolved Fe in the Southern Ocean (Fourquez et al., 2015, 2020; Mazzotta et al., 2020).

Theoretically, two species competing for the same limiting resource cannot coexist at constant population levels (Hardin, 1960). However, colimitation can lead to stable coexistence (Burson et al., 2018) or alternative stable states, depending on ambient environmental factors (e.g., resource supply and lability, temperature, light, and mortality) and the traits of the competing species (Brauer et al., 2012; Tilman, 1982) that affect resource uptake and growth (Hutchinson, 1953; Titman, 1976). In the Southern Ocean, the distinct seasonal light cycle regulates the timing of phytoplankton blooms, whereas Fe controls the magnitude of growth. While phytoplankton productivity then fluctuates according to season and location (Ardnya et al., 2017), the implications of changes in Fe, DOC, and light over the seasonal cycle on phytoplankton‐bacterial interactions are poorly understood. Upwelling of deep water is generally considered to supply a relatively refractory pool of DOC (Hansell, 2013). However, the release of labile DOC via phytoplankton exudation (Larsson & Hagström, 1979; Wood & Van Valen, 1990) or by other biotic processes commonly linked to phytoplankton production, such as sloppy feeding and fecal production by predators (Lampitt et al., 1990; Møller, 2007; Møller et al., 2003), viral lysis (Bratbak et al., 1993; Gobler et al., 2003; Middleboe et al., ; Poorvin et al., 2004), and phytoplankton cell mortality (Veldhuis et al., 2001) suggests that seasonal changes in phytoplankton production may facilitate phytoplankton and bacterial coexistence in the Southern Ocean by supplying labile DOC. While labile DOC is often rapidly utilized within hours (Fourquez et al., 2014; Obernosterer et al., 2015), seasonal accumulation of DOC has been observed in the Bermuda Atlantic Time Series site possibly due to phosphorus limitation of bacterioplankton growth (Cotner et al., 1997; Rivkin & Anderson, 1997) or the accumulation of recalcitrant DOC that prevents rapid utilization (Carlson & Ducklow, 1996; Carlson et al., 1998; Cherrier et al., 1996; Legendre & Le Fevre, 1995; Søndergaard et al., 2000). The turnover time for recalcitrant DOC ranges from months (semilabile) to 40,000 years (ultrarefractory) (Carlson & Ducklow, 1995; Cherrier et al., 1996; Hansell, 2013; Hansell et al., 2012; Lønborg et al., 2018).

At a cellular level, phytoplankton can use mechanisms such as Fe storage protein (i.e., ferritin) and vacuolar storage that enable them to stockpile Fe for use when ambient Fe levels are low (e.g., Cohen et al., 2018; Lampe et al., 2018; Marchetti et al., 2009). A more limited set of observations indicates that although bacteria possess two types of ferritin‐like molecules, the bacterial ferritins and bacterioferritin (Andrews et al., 2003; Debeljak et al., 2019; Rivera, 2017), the regulation of the bacterioferritin gene is unclear (Carrondo, 2003). Nevertheless, the higher Fe quotas observed in bacteria, relative to phytoplankton (Boyd et al., 2012; Fourquez et al., 2015, 2020; Mazzotta et al., 2020; Sarthou et al., 2008; Tortell et al., 1996), suggest that bacteria may require more Fe, or have the capacity for luxury Fe uptake, and hence may compete with phytoplankton for Fe. The parallel impact of other environmental regulatory factors, such as light or labile DOC, on the outcome of such a competition is unclear.

In this study, we develop a mechanistic model to examine the competitive interactions between phytoplankton and bacteria in response to Fe, light, and DOC colimitation. Our model was designed to assess the mechanisms behind these competitive interactions in an idealized setting and to explore the implications of these interactions on seasonal bloom dynamics in the Southern Ocean. We find that resource competition influences the magnitude of phytoplankton and bacterial biomass and rates of Fe uptake, growth, and carbon fixation—which depend on secondary factors affecting the growth and biomass accumulation of phytoplankton and bacteria, such as light, DOC, and luxury Fe uptake.

## 2 Methods

### 2.1 Biogeochemical Model

**Figure 1:**
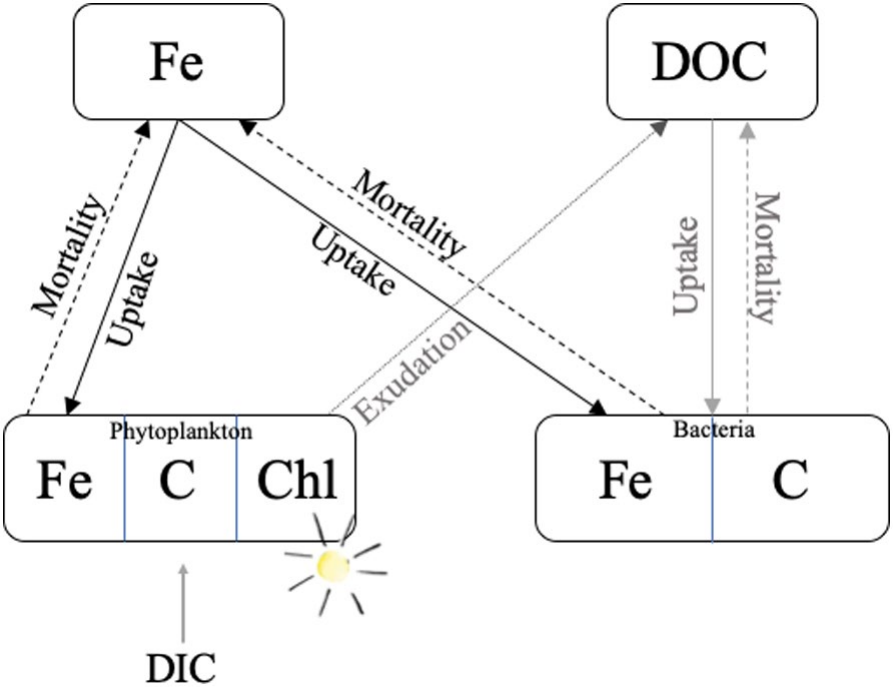
Schematic of model configuration with two biological pools: phytoplankton and bacteria, and three resources: dissolved iron (Fe), light, and dissolved organic carbon (DOC). Fe and DOC are taken up by phytoplankton and bacteria (solid lines) and recycled via mortality (dashed lines) and phytoplankton exudation (dotted).

The rate of change in phytoplankton carbon, chlorophyll‐a, and dissolved Fe is based on the Pelagic Integration Scheme for Carbon and Ecosystem studies (PISCES‐v2, Aumont et al., 2015) model, which is commonly used as part of global studies of ocean Fe biogeochemistry (e.g., Tagliabue et al., 2016). Here, we also explicitly account for the change in bacterial carbon and Fe biomass. Changes in phytoplankton and bacterial carbon and Fe biomass, as well as phytoplankton cellular chlorophyll‐a, are driven by rates of growth, carbon fixation, chlorophyll synthesis, and Fe uptake in response to Fe, light (modeled here as the photosynthetic available radiation, PAR), and labile DOC (Figure 1). We identify two sources of labile DOC: phytoplankton exudation and additional biotic sources which can be supplied via grazing processes (excretion, sloppy feeding), viral lysis, etc. Detailed equations and specific parameterizations are described in the supplementary material (Supplementary Methods and Table S1). Here, we briefly describe the underlying processes considered and their dependencies.

Dissolved Fe uptake by both phytoplankton and bacteria is modeled as a function of external Fe availability, including luxury Fe uptake and is downregulated when maximum cellular Fe quotas are reached. For simplicity, we assume phytoplankton and bacteria have the same maximum cellular Fe quota of 80 µmol Fe:mol C. This is based on the maximum phytoplankton cellular Fe quota used in PISCES‐v2 (Aumont et al., 2015) and maximum bacterial cellular Fe quota from a laboratory experiment under Fe‐replete conditions (Mazzotta et al., 2020). Labile DOC uptake by bacteria is modeled as a function of the temperature‐dependent maximum specific growth rate and bacterial Fe limitation. Light limitation of phytoplankton is identical to the PISCES‐v2 model explicitly accounting for phytoplankton carbon production and chlorophyll synthesis. Resource limitation for both phytoplankton and bacteria follow the quota model approach for Fe used in PISCES‐v2 and a Monod style limitation form for labile DOC. Phytoplankton carbon fixation is then a function of the temperature‐dependent maximum growth rate and light and Fe limitation. DOC is cycled via exudation and mortality and Fe is cycled via mortality (Figure 1). DOC exudation is a fixed proportion of phytoplankton carbon fixation (10%; Aumont et al., 2015). A quadratic mortality drives the recycling of cellular Fe, carbon, and chlorophyll.

### 2.2 Experimental Design

Using our model, we explore the role of Fe‐light and Fe‐DOC coregulation of phytoplankton and bacteria using two sets of model simulations at a constant temperature of 1°C. We first run a set of simulations with phytoplankton alone, across a range of light and Fe levels (from 1 to 40 W m^−2^ and 0 to 1 nM for PAR and Fe, respectively) and bacteria alone, across a range of labile DOC and Fe levels (from 0 to 100 µM and 0 to 1 nM for total DOC and Fe, respectively). These initial experiments allow us to explore the separate responses of phytoplankton and bacteria to changing light, Fe, and DOC levels. We then run a second set of experiments with both phytoplankton and bacteria competing under varying light and Fe (Fe‐PAR), with a fixed DOC level of 5 µM (broadly representative of typical labile DOC levels, Hansell et al., 2009), and varying Fe and DOC (Fe‐DOC), with a fixed light level of 20 W m^−2^ (broadly representative of typical mixed layer average Southern Ocean light levels). To specifically address the potential role played by luxury Fe uptake in bacteria, we run a parallel simulation with variable cellular Fe quota for phytoplankton but fixed the Fe quota for bacteria at a constant 10 µmol Fe:mol C (Aumont et al., 2015). We use the model results after 35 days of integration (by which time the model has reached quasi steady state) in our analysis.

## 3 Results and Discussion

### 3.1 Response to Resource Colimitation in the Absence of Competitive Interactions

**Figure 2:**
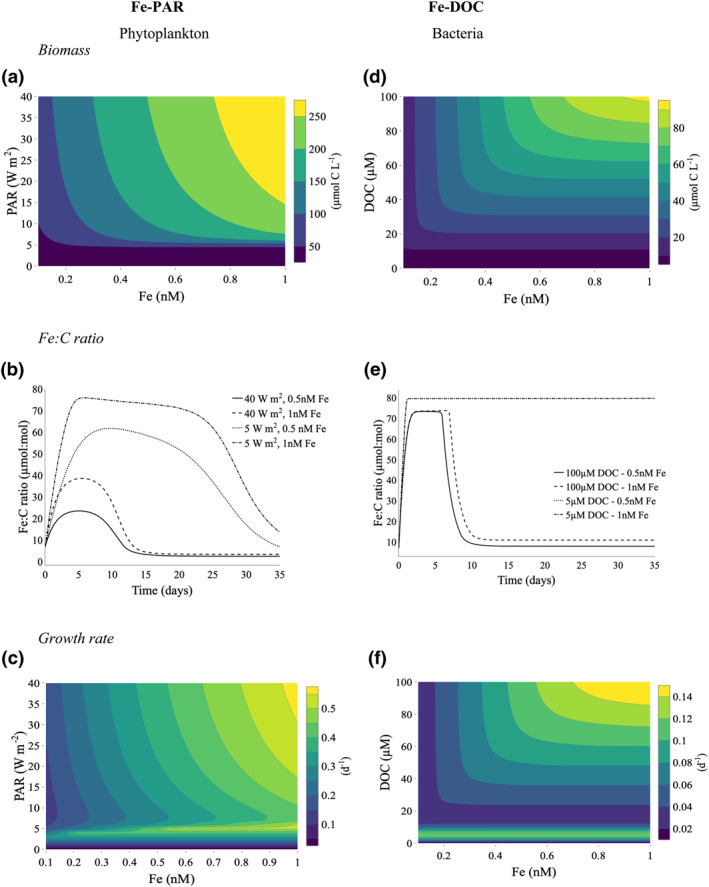
Steady state response of phytoplankton under iron (Fe) and light (photosynthetic available radiation) colimitation (a–c) and bacteria under Fe and dissolved organic carbon (DOC) colimitation (d–f) in the absence of competitive interactions. Changes in carbon biomass (a and d), cellular Fe quota (b and e), and growth rate (c and f). Note that the response of Fe:C ratios (e) in the 5 µM DOC, 0.5 nM Fe (dotted line) treatment is hidden behind the 5 µM DOC, 1.0 nM Fe (dot dash) treatment.

Increasing Fe and light leads to an expected increase in phytoplankton carbon biomass (Figure 2a), with the phytoplankton cellular Fe quota (Fe:C ratio) increasing as ambient Fe concentration increases (Figure 2b). However, as light increases in parallel to Fe, phytoplankton accumulate less cellular Fe relative to carbon because of high rates of growth and carbon fixation (Figures 2b and 2c). Increasing Fe and DOC similarly leads to an increase in carbon biomass of bacteria (Figure 2d). Although bacteria reach their maximum cellular Fe quota in all our simulations, bacterial growth remained restricted at low DOC concentrations due to DOC limitation which leads to high Fe:C ratios due to low rates of C assimilation (Figure 2e). Increasing Fe and DOC in parallel results in a sevenfold increase in bacterial growth rate (Figure 2f).

### 3.2 How Does Iron–Light Influence Phytoplankton–Bacterial Interactions?

**Figure 3:**
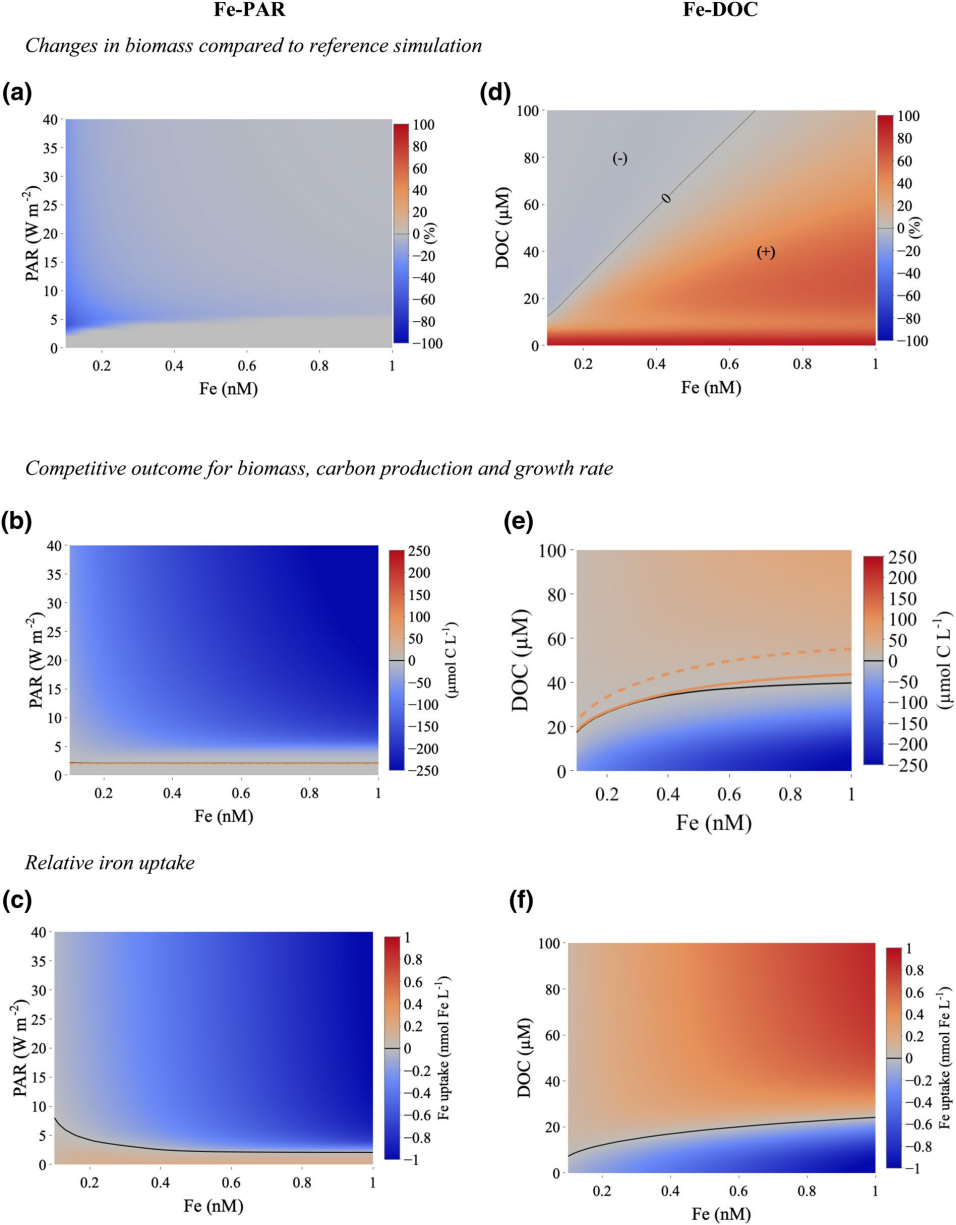
Steady state response of phytoplankton and bacteria as they compete for iron (Fe) under Fe‐light (PAR) (a–c) and Fe‐DOC colimitation (d–f). Carbon biomass relative to no competition simulation (a and d). Difference in biomass (black), growth rate (orange dash), and carbon production rate (orange solid) between phytoplankton and bacteria (b and e). Difference in Fe uptake rate between phytoplankton and bacteria (c and f). All lines denote regions where phytoplankton and bacteria are equal and separate regions of phytoplankton (blue) and bacterial (red) dominance.

Competition for Fe between phytoplankton and bacteria affects growth rates, production, and Fe uptake, which influences the relative accumulation of biomass. Under varying Fe and light, but fixed DOC, competition with bacteria decreases phytoplankton biomass by 3%–60% (Figure 3a) relative to the simulations with only phytoplankton due to bacterial Fe uptake, with greatest change observed at the lowest Fe levels across all light levels. We denote the 1:1‐line (i.e., region where phytoplankton biomass equals bacteria biomass), which separates regions of phytoplankton (blue) and bacterial (red) biomass dominance as the “line of coexistence.” This line of coexistence when defined in terms of carbon biomass is very similar to that defined from the specific growth rate (day^−1^, orange dash) and carbon production rate (orange solid) (Figure 3b), due to their fundamental role in driving biomass accumulation in our model. Below the line of coexistence (red area), bacteria are dominant, but their biomass remains low as low levels of phytoplankton productivity result in insufficient DOC supply from phytoplankton exudation. Above this line of coexistence (blue area), increasing light and Fe increases phytoplankton growth rate and carbon production resulting in overall phytoplankton dominance as they outcompete bacteria for Fe. As phytoplankton biomass increases, the DOC inventory increases via exudation, which stimulates bacterial growth in parallel, but bacterial biomass remains less than phytoplankton biomass (Figures S1a and S1b). This is because the exudation of DOC by phytoplankton at the start of the experiment is insufficient for bacteria to outcompete phytoplankton. The low DOC levels allow phytoplankton to respond more strongly in general by increasing growth rates, carbon production, and biomass (Figures S1a, S1b, S2a, and S2b).

The difference in the rates of Fe uptake between phytoplankton and bacteria (Figure 3c) shows a slightly different form to that seen for biomass, growth, and carbon fixation at low Fe and light. Bacteria consume up to 50% of the ambient Fe pool at low Fe (<0.2 nM) and light (<8 W m^−2^) levels due to low phytoplankton biomass levels and higher Fe uptake efficiency (i.e., lower bacterial half saturation constant for Fe uptake, Table S1), but increasing Fe and light causes greater phytoplankton biomass to accumulate (Figure 3c). We find that phytoplankton can outcompete bacteria for Fe uptake at high light and Fe (Figure 3c) due to their higher biomass and growth rate (Figures S1a, S2a, and S2b). In contrast, when bacteria rely solely on phytoplankton exudation as a DOC source, bacteria become relatively poor competitors for available Fe across these experiments.

### 3.3 How Does Iron–DOC Influence Phytoplankton–Bacterial Interactions?

Under varying Fe and DOC, but fixed light, bacteria receive two sources of DOC; DOC from phytoplankton exudation and DOC from additional biotic processes. In the natural environment, zooplankton excretion, viral lysis, and sloppy feeding represent additional biotic sources of DOC as well as Fe. While there are limited quantitative estimates on the contribution from each component, Antarctic krill were shown to excrete 188–560 µM labile DOC that was rapidly utilized by the bacterial community within the 140–160 h experimental duration leading to an increase in bacterial biomass and production (Arístegui et al., 2014).

In contrast to the Fe‐PAR simulation (where the only DOC was from phytoplankton exudation), phytoplankton–bacterial competition increases bacterial biomass (“+”, Figure 3d) more substantially (by up to 90% at low DOC levels), relative to the simulations with bacteria alone. The increase in bacterial biomass is driven by high phytoplankton biomass, growth rates and carbon production (Figures S1c, S1d, and S2d) which adds to the DOC inventory via phytoplankton exudation thereby facilitating bacterial growth (Figures 3d and 3e). An exception is at high DOC and low Fe where competitive Fe uptake by phytoplankton results in a small (<6%) decrease in bacterial biomass (“−”, Figure 3d). Greater DOC levels increasingly shift the dominance from phytoplankton to bacteria throughout this experiment, pointing to the importance of DOC in driving the competitive fitness of bacteria. The biomass line of coexistence is similar to the 1:1 line for carbon production (orange solid), but phytoplankton growth rate at this point is ∼10% greater than bacterial growth rate (Figures 3e and S2). Fe uptake is decoupled from biomass levels and carbon production rates (Figure 3f vs. Figure 3e). Bacteria consume between 50% and 80% of the ambient Fe pool except at low DOC (Figure 3f). While the Fe–PAR simulation suggests that bacteria are poor competitors for available Fe when they rely solely on phytoplankton exudation as a source of DOC, we find that bacteria are more effective competitors for Fe as total (phytoplankton exudation + additional biotic processes) DOC increases.

At the extremes, our simulations show phytoplankton dominance under increasing light and Fe (Figures 3b, S1a, and S3b), but bacterial dominance under increasing total DOC and decreasing light (Figures 3e, S1b, S1d, S3a, and S3b). We find that under moderate light levels (20 W m^−2^), coexistence between phytoplankton and bacteria can be facilitated when labile DOC increases in tandem with Fe up to around 20–30 µM DOC. However, stable coexistence is only found in our simulations at low biomass levels where colimitation constrains both phytoplankton and bacterial populations (Figures 3b and 3e). Overall, our simulations show that in the presence of sufficient labile DOC, bacteria are effective competitors with phytoplankton for Fe, exacerbating phytoplankton Fe limitation, most notably under moderate light levels.

Our model suggests that bacteria are an important consumer of Fe (Figures 3c and 3f), in line with the field studies that demonstrated higher Fe uptake by bacteria compared to phytoplankton (Boyd et al., 2012; Fourquez et al., 2015, 2020; Sarthou et al., 2008; Tortell et al., 1996). Furthermore, we find that bacteria are unable to sustain high growth rates in the absence of luxury Fe uptake (Figure S4). If phytoplankton were the only organisms undertaking luxury Fe uptake in our model, then phytoplankton would outcompete bacteria everywhere above the lowest light levels (2 W m^−2^) required to sustain phytoplankton growth, even at very high labile DOC levels (100 µM) as phytoplankton are able to rapidly consume and store any available Fe (Figures S4a–S4d). This highlights the importance of bacterial Fe storage in shaping the competition between phytoplankton and bacteria for Fe and is consistent with other approaches based on metalloproteins (Mazzatto et al., 2020).

### 3.4 Impact of Covariance in Fe, PAR, and DOC Over the Southern Ocean Seasonal Cycle

**Figure 4:**
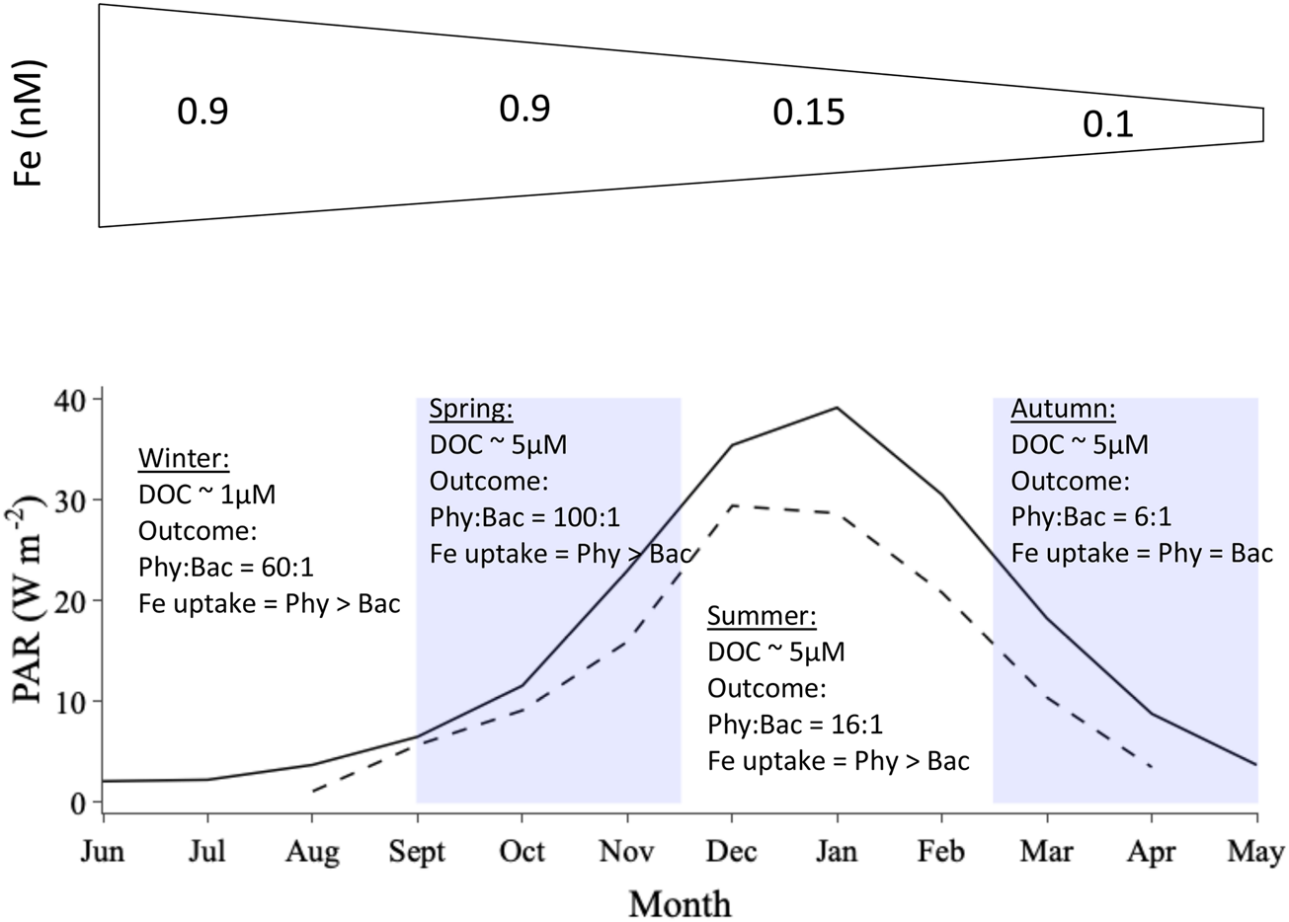
Top panel represents dissolved iron (Fe) fluxes from observations and modeling studies. Bottom panel demonstrates average mixed layer depth integrated light (photosynthetic available radiation; based on VIIRS Ocean Color Data, Ocean Biology Processing Group [2019] and de Boyer Montegut et al. [2004]) for the subantarctic (solid line) and polar (dashed line) zones and assumptions of labile DOC available for bacteria used to initialize the model. Highlighted rectangular regions represent austral spring and autumn.

There are two distinct regions within the Southern Ocean; the subantarctic zone (SAZ) and polar zone (PZ) which each encounters four different seasonal regimes; winter (June–August), spring (September–November), summer (December–February), and autumn (March–May). The availability of Fe, DOC, and PAR is likely to vary between these four regimes and between the SAZ and PZ. We use satellite estimates of PAR (NASA Goddard Space Flight Center, Ocean Ecology Laboratory, Ocean Biology Processing Group, 2019) coupled with Fe fluxes from observations and modeling studies (Figure 4) and the assumption that labile DOC exudation is ∼10% of phytoplankton primary production, to initialize our model and examine the strength of phytoplankton–bacterial interactions in each seasonal regime.

In winter, deep mixed layers and ice cover result in complete darkness in the PZ, while PAR levels remain around 2–7 W m^−2^ in the SAZ (Figure 4). As the supply of deep Fe will be maximal at this time, dissolved Fe levels in seawater will be expected to reach their seasonal maxima of around 0.9 nM in the surface open ocean (Tagliabue et al., 2014) and perhaps >1 nM closer to the shelf (Tagliabue et al., 2012). At this time of year, there is only a deep source of refractory DOC in large areas of the open ocean (Hansell, 2013). Under these conditions, we find that phytoplankton and bacterial biomass are low in the SAZ where there is low light. Yet the very low biomass of phytoplankton take up ∼98% of the Fe pool and deplete its concentration from 0.9 to 0.3 nM only after 35 days. Over this time, phytoplankton biomass can increase to from 0.1 to 6 µmol C L^−1^ but we observe no sustained increase in bacterial biomass despite a build‐up of 1 µM of DOC in our experiment from phytoplankton exudation. Phytoplankton outcompeted bacteria because the low labile DOC at the onset of the experiment constrained bacterial growth and instead allowed phytoplankton to drawdown Fe and moderately increase in biomass despite the apparent light limitation. To maintain seawater dissolved Fe levels of >0.7 nM throughout winter in the SAZ, either Fe must be continuously supplied via deep winter mixing (Tagliabue et al., 2014) or >50% of the particulate Fe in phytoplankton and bacteria needs to be rapidly recycled. In the PZ, despite the darkness, grazing by the resident zooplankton community (e.g., Antarctic krill, Walsh et al., 2020) under the winter ice can lead to the coupled release of Fe and DOC potentially driving competition between bacteria and low‐light acclimated ice algae, a community of phytoplankton not modeled in this study.

In spring, PAR levels increase to 20 W m^−2^ in the PZ and 30 W m^−2^ in the SAZ (Figure 4). Shoaling of the mixed layer maintains high dissolved Fe stock of ∼0.9 nM at the onset of spring (Tagliabue et al., 2014). Initializing our model under these high Fe and light levels, coupled with the 1 µM of DOC that built up over the winter period, we find that phytoplankton outcompete bacteria at a ratio of 100:1 and consume >98% of the dissolved Fe pool within 13 (PAR 30 W m^−2^) to 15 (PAR 20 W m^−2^) days because the low DOC at the onset of spring allows phytoplankton to outcompete bacteria. PAR continues to increase to the seasonal maximum of 30 W m^−2^ in the PZ and 40 W m^−2^ in the SAZ during summer, but dissolved Fe concentrations tend to decrease to ∼0.15 nM in the surface open ocean as phytoplankton have depleted the winter stock and recycling turns over the dissolved Fe stock rapidly (Boyd et al., 2012; Strzepek et al., 2005; Tagliabue et al., 2014). Under low Fe and high light conditions, and assuming a labile DOC stock of ∼5 µM in addition to continual DOC exudation (10% of primary production) (Hansell et al., 2009), phytoplankton take up ∼80% of the ambient Fe and phytoplankton biomass ∼16 times greater than bacteria. From late summer into autumn, the mixed layer starts to deepen, PAR decreases from 20 to 0 W m^−2^ (Figure 4) and Fe is at its seasonal minimum (∼0.1 nM, Tagliabue et al., 2012). Here, phytoplankton take up ∼55% of the Fe and phytoplankton biomass is ∼6 times greater than bacteria.

The low standing stocks of Fe in spring/summer do not preclude fast recycling via bacterivory and herbivory coupled with rapid utilization (Strzepek et al., 2005). Bacterivory and herbivory would lead to the coupled release of Fe and DOC. If we assume greater levels of Fe and labile DOC, that might be representative of the greater recycling (e.g., bacterivory; Strzepek et al., 2005), then the competitive fitness of bacteria is enhanced. Our results suggest that the amount of DOC necessary to switch the dominance from phytoplankton to bacteria varies as a nonlinear function of Fe (black line in Figure 3e). At the low Fe levels, typical of summer as little as 20 – 30 µM DOC would alter the competitive outcome, while at higher Fe levels typical of earlier in the bloom phase more DOC is needed (Figure 3e).

Our analysis suggests that decreasing light and Fe, coupled with a small DOC inventory (5 µM), facilitates Fe uptake by bacteria, but microbial Fe uptake is decoupled from carbon production. Phytoplankton biomass is consistently greater than bacterial biomass across all seasons. Bacteria require >50 µM of labile DOC to outcompete phytoplankton biomass in spring. This decreases to 40 µM in summer, 15 µM in autumn, and 8 µM in winter. Bacteria require less DOC as the season progresses because of the colimiting effects of Fe and light on phytoplankton growth. This suggests that sources such as ice, dust, sediments, and recycling with varying Fe and DOC levels may influence different components of the microbial community, with the strength of the interaction further compounded by seasonal effects on phytoplankton growth.

## 4 Competition Under Changing Climate: Uncertainties and Future Work

There is a strong interest in understanding the response of phytoplankton to a changing climate due to its role in the global carbon cycle and supporting ocean fisheries. Southern Ocean surface seawater is predicted to warm by 2–3°C by the year 2100. Both phytoplankton and bacterial metabolism respond positively with an increase in temperature (Deppeler & Davidson, 2017; Pomeroy & Wiebe, 2001; Rivkin et al., 1996; Toseland et al., 2013). However, there is substantial disagreement on how temperature could regulate phytoplankton‐bacterial interactions and DOC production and stimulation. Bacterial production could follow an increase in primary production and not temperature (Kirchman et al., 1995; Morán et al., 2006) or respond to an increase in temperature and not primary production (Hoppe et al., 2008). Our analysis finds that bacteria can outcompete phytoplankton leading to a decrease in phytoplankton biomass if sufficient labile DOC is available which can be found in regions of high recycling (Arístegui et al., 2014; Ruiz Halpern et al., 2011) and ice melt (Norman et al., 2010; Underwood et al., 2010), where there is also concurrent release of Fe, a concept that has previously not been considered in our understanding of the Fe cycle in the Southern Ocean (Tagliabue et al., 2014). As ice melt increases with rising temperatures, the increase in Fe and DOC supply coupled with an increase in temperature may stimulate bacterial communities at the expense of phytoplankton. Furthermore, increased temperature can lead to increased DOC exudation by phytoplankton (Zlotnik & Dubinsky, 1989) and greater microbial degradation of recalcitrant DOC (Lønborg et al., 2018), which suggests the potential for greater DOC availability under warmer climate. However, ice melt can strongly stratify the surface water, thereby relieving phytoplankton light limitation and strengthening competition with bacteria.

Additional feedbacks, not explored in this study, are the interactions around the bacterial production of siderophores in response to Fe scarcity (e.g., Boiteau et al., 2016) and the weaker Fe binding agents associated with phytoplankton exudates, such as saccharides (Hassler et al., 2011) or heme groups (Louropoulou et al., 2020), or the role of bacterivory in recycling the Fe stored in bacteria (Boyd et al., 2012, 2015; Mazzotta et al., 2020; Strzepek et al., 2005; Tortell et al., 1996). Furthermore, along the coastal Antarctic sea‐ice edge communities, phytoplankton are limited by cobalamin (vitamin B_12_) and Fe (Bertrand et al., 2015). As cobalamin is only produced by bacteria and archaea, this suggest that phytoplankton‐bacterial interactions mediate micronutrient colimitation for nutrients other than Fe as well. As such, more experimental‐modeling work is required to gain a better mechanistic understanding of how the competitive interactions between phytoplankton and bacteria could change in response to multiple concurrent environmental changes.

## 5 Conclusions

Overall, we find that resource colimitation and competition alters the response of phytoplankton and bacteria. Phytoplankton growth rate decreases due to competition for Fe uptake by bacteria. In contrast, the increase in DOC supplied via phytoplankton exudation leads to an increase in bacterial growth rate but is insufficient to stimulate bacterial biomass to an extent that bacteria outcompete phytoplankton. If additional DOC is supplied, for instance from recycling or sea ice melting, then bacteria can successfully outcompete phytoplankton for Fe and depress rates of phytoplankton productivity. As large areas of the Southern Ocean are characterized by low dissolved Fe‐low labile DOC, the seasonal cycle of light and ensuing exudation, grazing, viral lysis, and cell death which increases DOC inventory ultimately regulates phytoplankton‐bacterial interactions.

## Supporting information

Supporting Information S1

## Data Availability

The model used in this study is based on the global 3D biogeochemical model PISCES as described in Aumont et al. (2015) and freely available as part of the NEMO modeling platform. The NEMO code is available from https://www.nemo-ocean.eu. The equations and parameters used in this study are provided in the supplementary material. Mixed layer depth data were sourced from de Boyer Montegut et al. (2004) (http://www.ifremer.fr/cerweb/deboyer/mld/Surface_Mixed_Layer_Depth.php) and PAR was sourced from NASA Ocean Color (VIIRS monthly climatological data at 4‐km resolution https://oceancolor.gsfc.nasa.gov/l3/).
