# Supplementary material for: Resource Colimitation Drives Competition Between Phytoplankton and Bacteria in the Southern Ocean
Source: Geophys Res Lett. 2021 Jan 12;48(1):e2020GL088369. doi: 10.1029/2020GL088369 (PMC7816276; doi:10.1029/2020GL088369)
Supplement: Supplementary file 1 — Supporting Information S1 [file GRL-48-e2020GL088369-s001.pdf]

Supporting Information for

Resource colimitation drives competition between phytoplankton and bacteria in the Southern Ocean

Lavenia Ratnarajah[1], Stéphane Blain[2], Philip W. Boyd[3], Marion Fourquez[4,3], Ingrid Obernosterer[2], Alessandro Tagliabue[1]

1. Department of Earth, Ocean and Ecological Sciences, School of Environmental Sciences, University of Liverpool, Liverpool, United Kingdom
2. Sorbonne Université, CNRS, Laboratoire d'Océanographie Microbienne (LOMIC), Observatoire Océanologique de Banyuls, 66650 Banyuls sur mer, France
3. Institute for Marine and Antarctic Studies, University of Tasmania, Tasmania, Australia
4. Aix Marseille Univ., Universite de Toulon, CNRS, IRD, MIO UM 110, 13288, Marseille, France

Contents of this file

Table S1

Figures S1 to S4

Supplementary methods

Supplementary table

Table S1: Summary of model parameters. Where two values are given, the first is for phytoplankton and the second is for bacteria.

**Parameter**	**Units**	**Value**	**Description**
δ	-	0.1	Exudation of DOC
m	d^{-1}	0.01 / 0.01	Maximum quadratic mortality of phytoplankton and bacteria
μ_{max}^{0}	d^{-1}	1 / 1	Growth rate at 0°C
b_P/b_B	-	1.066 / 1.09	Temperature sensitivity of growth
α^P	W $(m^{-2})^{-1}$ d^{-1}	2	Initial slope of P-I curve
θ^{Chl}	mg Chl (mg $C)^{-1}$	0.01	Chlorophyll to carbon ratios of phytoplankton
b_{resp}	d^{-1}	0.0033	Basal respiration rate
$\theta_{min}^{Fe,B}$	µmol Fe (mol $C)^{-1}$	7	Minimum iron quota in bacteria
θ_{Opt}^{Fe}	µmol Fe (mol $C)^{-1}$	7 / 7	Optimal iron quota
θ_{max}^{Fe}	µmol Fe (mol $C)^{-1}$	80 / 80	Maximum iron quota
K_{DOC}	µmol C L^{-1}	0.5	Half saturation constant for dissolved organic carbon

K_{Fe}^{P}	nmol Fe L^{-1}	2	Half saturation constant for iron uptake by phytoplankton
K_{Fe}^{B}	nmol Fe L^{-1}	0.01	Half saturation constant for iron by bacteria

Supplementary figures

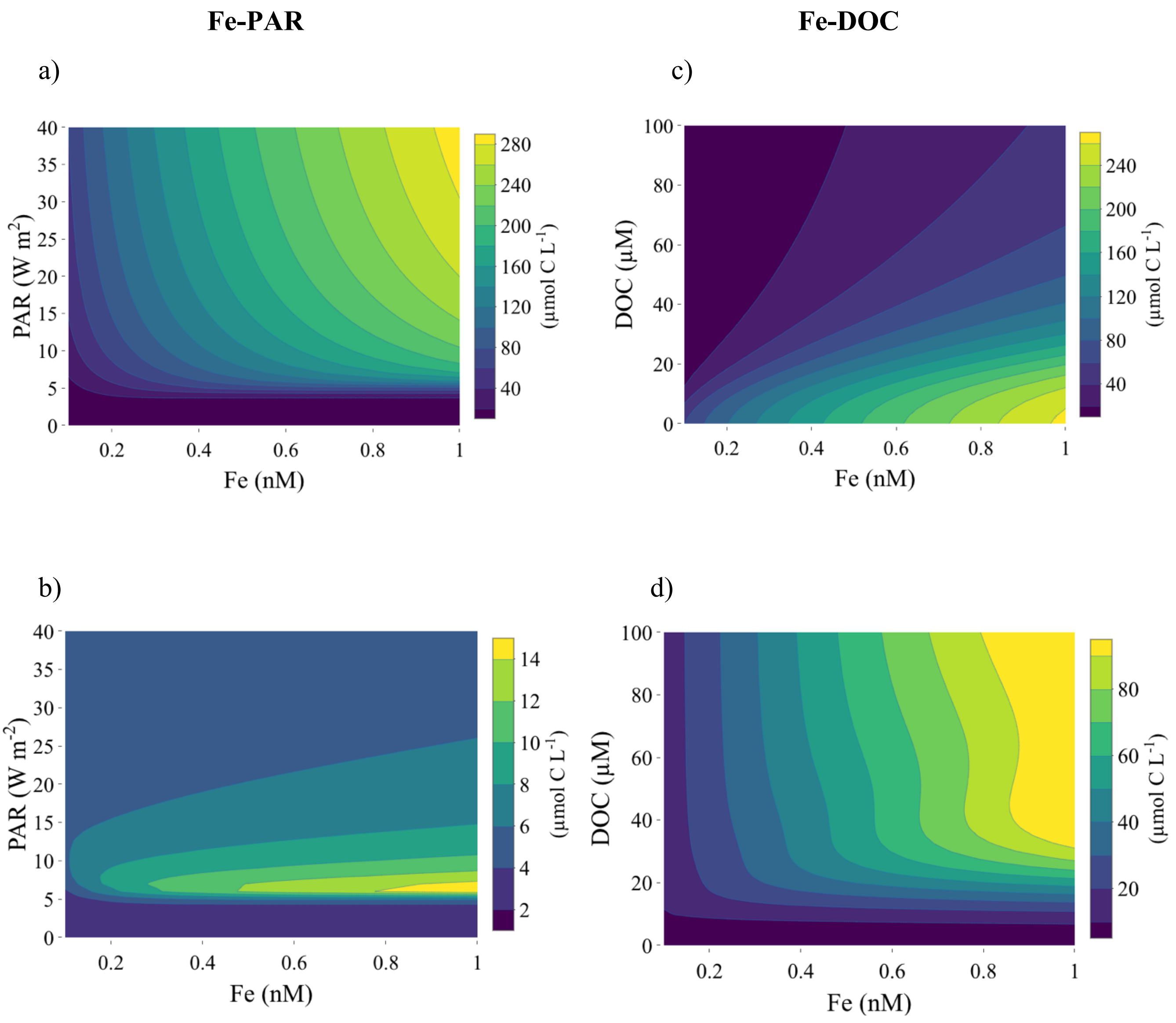

Figure S1: Steady state response of phytoplankton (a, c) and bacterial (b, d) biomass under iron (Fe) and light (PAR) (a, b), and Fe and dissolved organic carbon (DOC) colimitation (b, d).

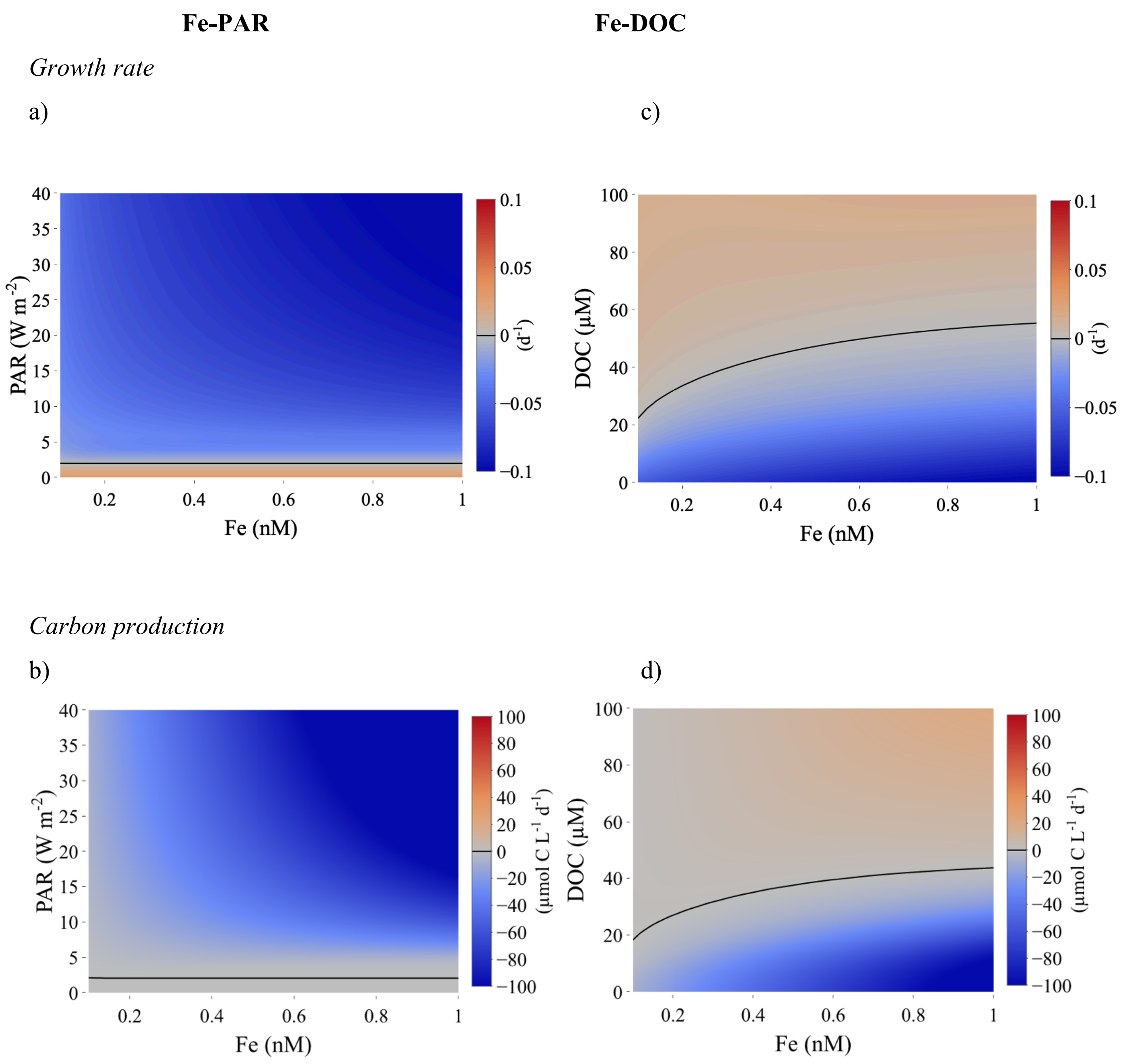

Figure S2: Competitive outcome between phytoplankton and bacterial growth rate (a, c) and carbon production (b, d) under iron (Fe) and light (PAR) (a, b), and Fe and dissolved organic carbon (DOC) (c, d) colimitation. The solid black line denotes region where phytoplankton growth rate (a, c) and carbon production (b, d) equals bacteria, and separates regions of phytoplankton (blue) and bacterial (red) dominance.

Fe-PAR **Fe-DOC**

a) b)

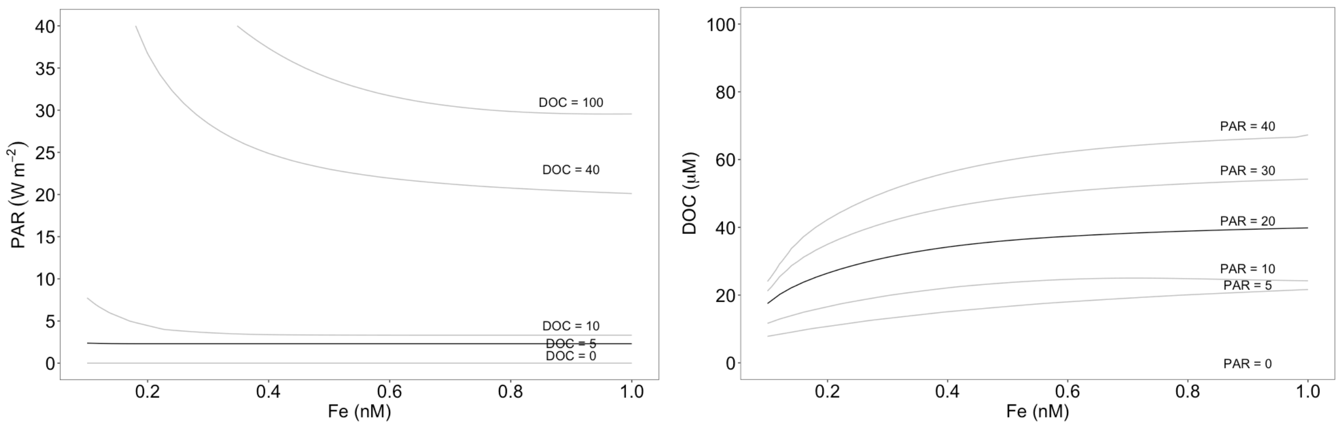

Figure S3: Steady-state response of phytoplankton and bacteria under iron (Fe) and light (PAR) colimitation with varying DOC levels (a), and Fe and dissolved organic carbon (DOC) colimitation with varying PAR levels (b). All solid lines denote regions where phytoplankton and bacteria equally constitute 50% of the total biomass. The black solid line in each panel represents the original model simulation.

Fe-PAR **Fe-DOC**

Competitive outcome for biomass, carbon production and growth rate

a)

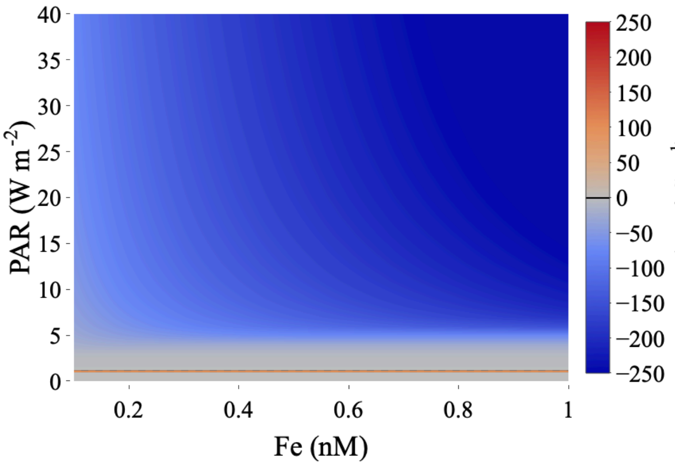

c)

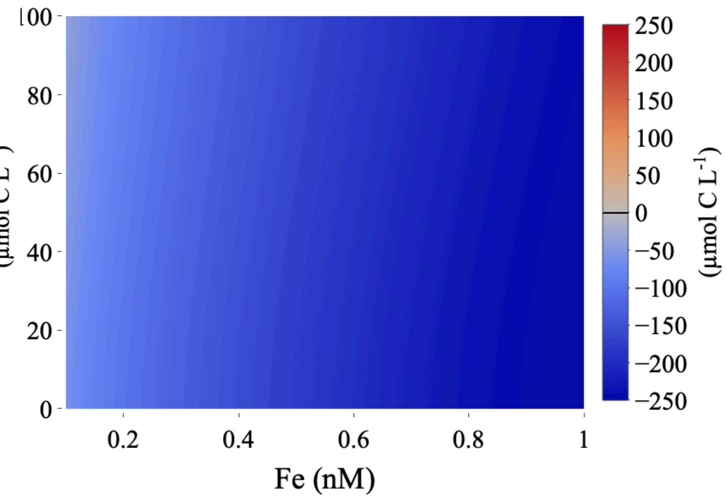

Relative Fe uptake

b)

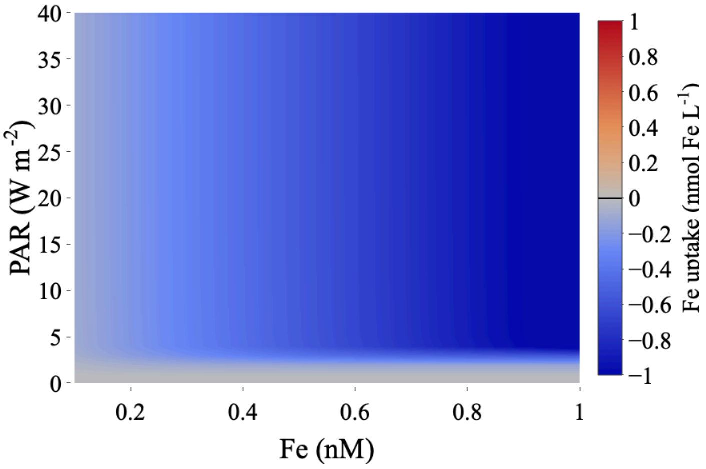

d)

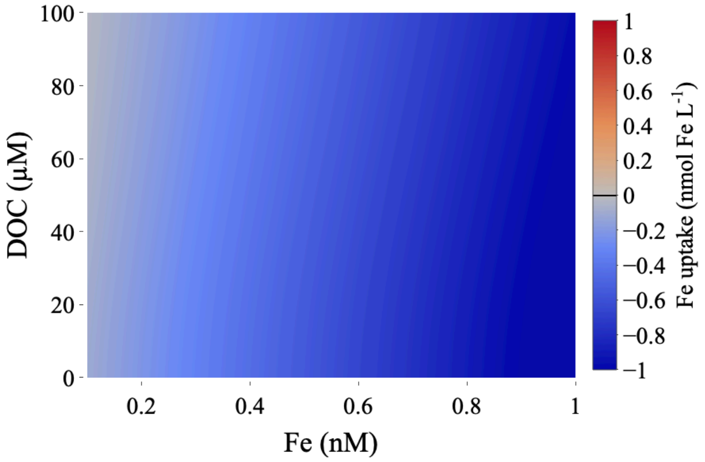

Figure S4: Biomass of phytoplankton and bacteria under iron (Fe) and light (PAR) (a, b) and Fe and dissolved organic carbon (DOC) (c, d) colimitation but accounting for luxury Fe uptake only in phytoplankton (i.e. no luxury Fe uptake by bacteria).

Supplementary methods

Model design

<u>Changes in biomass</u>

The strength of phytoplankton-bacterial interactions is quantified by the rate of change in phytoplankton (P) and bacterial (B) biomass (μmol C L^{-1}) in response to Fe, light (modelled here as the photosynthetic available radiation, PAR) and DOC:

$$\frac{\partial P}{\partial t} = (1 - \delta^P)\,\mu^P P - m^P * P \qquad \text{(Eq. 1)}$$

$$\frac{\partial B}{\partial t} = \mu^B P - m^B * B \qquad \text{(Eq. 2)}$$

where δ^P represents DOC exudation by phytoplankton, $\mu^P$and $\mu^B$are the growth rate of phytoplankton and bacteria respectively as a function of Fe, PAR and DOC and and are the quadratic mortality terms to avoid extinction at very low growth rates.

<u>Growth rate</u>

Phytoplankton growth rate is modelled based on PISCES-v2 (Equation 2a in Aumont et al. 2015) general equations:

$$\mu^P = \mu^0_{max}\, b_P\, f_1\,(L_{day})\; 1 - exp\, \frac{-\alpha^P \theta^{Chl} PAR}{b_{resp}\, L_{day}}\; L^P_{Fe(lim)} \qquad \text{(Eq. 3)}$$

The growth rate of phytoplankton is dependent on the temperature sensitivity of growth (b_P), the length of day ($f_1\,(L_{day})$), the slope of the photosynthetic-irradiance curve (α^P), chlorophyll-to-carbon ratio (θ^{Chl}), PAR and the availability of the limiting resource to meet metabolic function – Fe ($L^P_{Fe(lim)}$).

As bacterial growth rate is not dependent on light availability, their growth is computed as a function of sensitivity to temperature (b_B) and the availability of limiting resources; Fe ($L^B_{Fe(lim)}$) and DOC ($L^B_{DOC(lim)}$):

$$\mu^B = \mu_{max}^0 b_B (L_{Fe(lim)}^B \ x \ L_{DOC(lim)}^B) \qquad \text{(Eq. 4)}$$

Competition for Fe

$$L_{Fe} = min\ (1, max\ (0, \frac{\theta^{Fe} - \theta_{min}^{Fe}}{\theta_{opt}^{Fe}}) \qquad \text{(Eq. 5)}$$

The minimum Fe requirement for phytoplankton ($\theta_{min}^{Fe,P}$) is based on PISCES-v2 (Equation 20 in Aumont et al. 2015) and is dependent on the demand for photosynthesis, respiration and nitrate/nitrite reduction:

$$\theta_{min}^{Fe,P} = (\frac{0.0016}{55.85}\ \theta^{Chl}) + (\frac{1.21\ x\ 10^{-5}\ x\ 14}{55.845\ x\ 7.625}\ L_N^P\ x\ 1.5) + (\frac{1.15\ x\ 10^{-4}\ x\ 14}{55.845\ x\ 7.625}\ L_{NO3}^P)$$

Photosynthesis Respiration Nitrate/Nitrite reduction

(Eq. 6)

where nitrogen limitation (L_N^P) is modelled based on Monod parameterisation (Equation 6 in Aumont et al. 2015).

Relatively little is known about the fate of Fe in bacteria therefore making similar quantification of the metabolic demand for Fe in bacteria challenging. In this study the minimum and maximum cellular Fe quota in bacteria is fixed to match phytoplankton however bacterial growth can be co-limited by the availability and lability of DOC:

Based on the minimum Fe requirements determined above, phytoplankton (∂Q_P) and bacterial (∂Q_B) cellular Fe quota is quantified as:

$$\frac{\partial Q_P}{\partial t} = (1 - \delta^P)\ (Q_{max}^P\ x\ (\mu_{max}^0\ b_P)\ x\ \frac{Fe}{Fe + K_{Fe}^P}\ x\ max\ (0, \frac{1 - \frac{\theta^{Fe,P}}{\theta_{max}^{Fe,P}}}{1.05 - \frac{\theta^{Fe,P}}{\theta_{max}^{Fe,P}}})) -$$

$$(m\ x\ (P\ x\ \theta^{Fe,P})) \qquad \text{(Eq. 7)}$$

$$\frac{\partial Q_B}{\partial t} = (Q^B_{max} \; x \; (\mu^0_{max} \; b_B) \; x \; \frac{Fe}{Fe + K^B_{Fe}} \; x \; max\,(0, \frac{1 - \frac{\theta^{Fe,B}}{\theta^{Fe,B}_{max}}}{1.05 - \frac{\theta^{Fe,B}}{\theta^{Fe,B}_{max}}})) \; - \; (m \, x \, (B \, x \, \theta^{Fe,B}))$$

(Eq. 8)

Ambient nutrient concentrations

Fe (∂Fe) concentrations reflect the external supply of Fe and uptake by phytoplankton and bacteria whilst DOC (∂DOC) concentrations reflect the external supply of DOC, exudation by phytoplankton and uptake by bacteria. Phytoplankton and bacterial mortality allow for Fe and DOC to be recycled.

$$\frac{\partial Fe}{\partial t} = \frac{\partial P}{\partial t} + (-\frac{\partial B}{\partial t}) \qquad \text{(Eq. 9)}$$

$$\frac{\partial DOC}{\partial t} = \left((\delta^P \; x \; \mu^P) + (m \; x \; P)\right) + \left((\mu^B + (m \; x \; B)\right) \qquad \text{(Eq. 10)}$$
